# Usefulness of Ultrasound Imaging in Detecting Psoriatic Arthritis of Fingers and Toes in Patients with Psoriasis

**DOI:** 10.1155/2011/390726

**Published:** 2011-02-08

**Authors:** Clara De simone, Giacomo Caldarola, Magda D'Agostino, Angelo Carbone, Cristina Guerriero, Lorenzo Bonomo, Pierluigi Amerio, Nicola Magarelli

**Affiliations:** ^1^Department of Dermatology, Catholic University of the Sacred Heart, Largo Francesco Vito1, 00168 Rome, Italy; ^2^Department of Bioimaging and Radiological Sciences, Catholic University of the Sacred Heart, Largo Francesco Vito1, 00168 Rome, Italy

## Abstract

*Background*. Given that clinical evaluation may underestimate the joint damage and that early treatment can slow down psoriatic arthritis (PsA) progression, screening psoriasis patients with imaging tools that can depict early PsA changes would entail clear benefits. *Objective*. To compare the ability of X-ray and ultrasound (US) examination in detecting morphological abnormalities consistent with early PsA in patients with psoriasis, using rheumatological evaluation as the gold standard for diagnosis. *Methods*. Patients with chronic plaque psoriasis and no previous PsA diagnosis attending our outpatient dermatology clinic and reporting finger/toe joint and/or tendon pain underwent X-ray and US evaluation; they were subsequently referred to a rheumatologist for clinical examination and review of imaging findings. *Results*. Abnormal US and/or X-ray findings involving at least one finger and/or toe (joints and/or tendons) were seen in 36/52 patients: 11 had one or more X-ray abnormalities, including erosion, joint space narrowing, new bone formation, periarticular soft tissue swelling, and periarticular osteoporosis; 36 had suspicious changes on US. *Conclusion*. US proved valuable in detecting joint and/or tendon abnormalities in the fingers and toes of patients with suspicious changes. The dermatologist should consider US to obtain an accurate assessment of suspicious findings.

## 1. Introduction

Psoriatic arthritis (PsA) is a form of inflammatory arthritis associated with psoriasis. It affects 7% to 36% of psoriatic patients in different samples and up to 1% of the global population [1–8]. Moll and Wright originally described five clinical manifestations of PsA: predominantly distal interphalangeal joint disease, asymmetrical oligoarthritis, polyarthritis, spondylitis, and arthritis mutilans [9]. Recently, the classic spectrum has been extended to encompass a number of extra-articular clinical manifestations including enthesitis (tenderness, warmth, and/or loss of function at the insertion of Achilles tendon, plantar fascia, and lateral or medial epicondyle), tendonitis, tenosynovitis, and dactylitis (swelling of the entire digit or “sausage digit”) [10, 11]. Although PsA is commonly considered as a benign arthropathy, about 50% of patients go on to develop progressive arthritis, with joint function impairment or loss and even deformity [12–14]. 

PsA develops after the cutaneous disease in approximately 70% of patients, simultaneously in 10%–15%, and before psoriasis in 15%–20% of cases [3, 12]. Since, in most patients psoriasis predates PsA onset by about 10 years [12], the dermatologist has a crucial role in early diagnosis, since each visit offers the opportunity to assess the joint pain that can suggest PsA. Clearly, a close working relationship between dermatologist and rheumatologist is the best premise for prompt PsA diagnosis and treatment [3, 4]; however, referral of all psoriatic patients with musculoskeletal pain to a rheumatologist would be impractical. Given that clinical evaluation may underestimate the joint involvement in these patients [15] and the high prevalence of undiagnosed cases of active PsA that has been reported among psoriasis patients followed by dermatologists [16], their screening with imaging tools that can depict early PsA changes would be very helpful. Such screening would identify those patients needing rheumatological evaluation, while allowing immediate initiation of treatment for the joint condition, as suggested by the rheumatologists [17]. 

Plain radiographs are the standard technique employed to document the typical proliferative and destructive bone lesions of PsA and are, therefore, very useful. However, they are insensitive to the soft tissue changes (peripheral joint effusion, synovium proliferation, structural abnormalities in tendons and entheses, tendon sheath thickening, and bursitis) that are the only signs of early arthritis [18]. Ultrasonography (US) with high-frequency probes (>10 MHz) is increasingly being used for its ability and sensitivity, compared with clinical evaluation and X-rays [19–22], in evaluating soft tissue involvement both in early inflammatory arthritis and in late disease [21–25]. Power Doppler sonography (PDS) affords visualization of small vessel flow, showing soft tissue inflammation and disease activity in peripheral arthritis [19, 26–32].

We compared the ability of X-ray and US examination to depict morphological changes consistent with PsA in the fingers and toes of psoriasis patients using rheumatological evaluation as the gold standard for diagnosis. Patients with chronic plaque psoriasis but without a diagnosis of PsA attending our Outpatient Psoriasis Clinic and reporting finger/toe pain were examined by X-rays and US and subsequently referred to a rheumatologist for clinical evaluation and assessment of the imaging findings. 

## 2. Material and Methods

### 2.1. Patients

Subjects were 52 consecutive outpatients with chronic plaque psoriasis and finger and/or toe pain (Group A) who were being followed at the Outpatient Psoriasis Clinic of Policlinico “A. Gemelli” (Rome, Italy). Inclusion criteria were age >18 years, a diagnosis of psoriasis for at least a year, and pain involving any finger and/or toe joint for >3 months. Exclusion criteria were a diagnosis of PsA or other inflammatory joint disease, current or recent (3 months) systemic treatment for psoriasis, a recent history of hand or foot trauma, and current engagement in heavy manual work. The clinical assessment included the count of finger and toe joints with joint-line tenderness, stress pain, and/or swelling. The joint count included the metacarpophalangeal (MCP), proximal and distal interphalangeal (PIP and DIP) joints of fingers, and the metatarsophalangeal (MTP) and interphalangeal (IP) joints of toes (with the proximal and distal interphalangeal joints of toes counting as one joint). The thumb IP joint was counted as a DIP joint. The presence of dactylitis was also recorded if “sausage digit” was observed. Patients underwent X-ray and US evaluation of fingers and toes within two weeks of the clinical assessment and were then referred to a rheumatologist for a definitive diagnosis.

Clinical and US evaluation of fingers and toes was also performed in 50 sex- and age-matched patients with chronic plaque psoriasis but no finger and/or toe joint pain meeting the same inclusion and exclusion criteria (Group B). In this group, abnormal US findings involved X-ray examination and referral to the rheumatologist. 

Age at psoriasis onset was recorded in both groups of patients. The severity of psoriasis was evaluated with the Psoriasis Area and Severity Index (PASI). 

### 2.2. Ultrasound and Power Doppler Examination

The US and PDS examination was performed by two musculoskeletal radiologists (N.M. and L.B.), who were unaware of patients' clinical and X-ray data. All joints and tendons of all fingers and toes were examined using a Toshiba Aplio XV machine with a multifrequency linear transducer (5–12 MHz), 500 Hz pulse repetition frequency, colour printer, and Doppler signal recording, independently of the site(s) of pain. Frequency was based on the joint being examined. The colour gain was set so as to prevent flash artefacts from stationary tissue. All MCP, PIP, DIP of fingers and MTP of toes were investigated; the PIP and DIP of toes were recorded as one joint per toe, as in the clinical evaluation. Longitudinal and transverse views from the dorsal and the palmar/plantar aspect were taken with the hand and foot in neutral position. Representative images of each joint were obtained in B mode and power Doppler mode. Flexor and extensor tendons of fingers and toes were studied in the same way. Joint effusion, synovial proliferation, tenosynovitis (effusion of tendon sheath), and erosion of bone contour on US were considered as being suggestive of PsA. Vascular spots in intra-articular or peritendinous spaces on PDS were considered as being suspicious for abnormal synovial tissue vascularity (inflammation). 

### 2.3. Radiographic Examination

Standard radiographs of hands and feet were obtained in dorso-palmar and oblique views and evaluated separately by the two radiologists, who were blind to the clinical and US/PDS data. In case of disagreement a consensus was reached by consulting a third radiologist. Erosion, joint space narrowing, new juxta-articular bone formation, periarticular soft tissue swelling, and periarticular osteoporosis were considered to be suggestive of PsA. 

## 3. Results

### 3.1. Psoriatic Patients with Finger and/or Toe Pain (Group A)

Patient's clinical characteristics are reported in Table 1 as mean ± SD. Group A patients were 30 men and 22 women (36.2 ± 9.6 years), who had a duration of the skin disease of 10.4 ± 8.2 years and a PASI score of 9.6 ± 11.5. On clinical evaluation, they had 0.8 ± 2.1 swollen and 2.3 ± 3.1 tender joints. One patient had dactylitis in one finger and three patients had dactylitis in one toe. 

**Figure 1:**
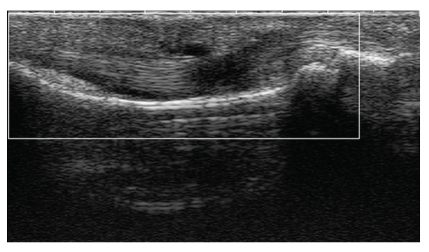
US scan. Index finger flexor tenosynovitis with fluid and thickening, suggesting PsA.

**Figure 2:**
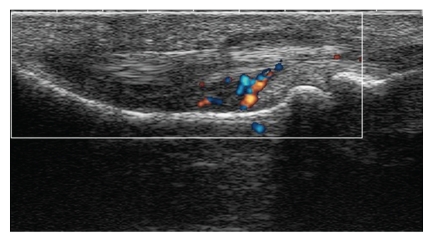
US-PDS scan. Index finger flexor tenosynovitis with fluid, thickening, and vascular spots.

Abnormal B mode (Figure 1) US findings suggestive of PsA in at least one finger and/or toe (joints and/or tendons) were seen in 36/52 patients. Twenty-nine of these 36 patients showed an increased vascularity on PDS in intra-articular and/or peritendinous spaces (Figure 2); 11 also had one or more X-ray abnormalities. In particular, tenosynovitis with vascular spots was detected in the flexor tendons of the affected digits in the 4 patients with dactylitis; in one of these patients, this was associated with effusion in a PIP joint. All 36 patients were diagnosed with PsA by the rheumatologist. The results of US, X-ray, and rheumatological evaluation of Group A subjects are summarized in Table 2. 

The 16/52 patients without findings suspicious for PsA were diagnosed by the rheumatologist with osteoarthritis (*n* = 11) and metabolic disease (*n* = 1); microtrauma pain was suspected in the remaining 4 patients. Six of these 16 subjects also had vascular spots on PDS. 

### 3.2. Psoriatic Patients without Finger and/or Toe Pain (Group B) 

The clinical characteristics of Group B subjects are reported in Table 1 as mean ± SD. They were 23 men and 27 women (age 32.1 ± 10.6 years) with a slightly but not significantly shorter disease duration (8.1 ± 7.5 years) compared with Group A patients. Their PASI score (7.9 ± 13.4) was comparable to that of Group A. None of these patients had dactylitis or any tender and/or swollen joint on clinical evaluation, or any finger and/or toe abnormality suggestive of PsA on US. Vascular spots on PDS examination were found in one toe joint in 3/50 subjects, and were not held to be related to a joint disease by the rheumatologist, but were interpreted as false positives depending on probe sensitivity or induced by microtrauma. However, 2 patients in this group were diagnosed with PsA based on concomitant axial involvement (not shown). 

## 4. Discussion

The heterogeneous clinical manifestations and course of PsA make diagnosis particularly elusive. Most patients experience progression of the clinical damage, while some 20% go on to develop a severe and debilitating form of arthritis [33, 34]. Since PsA arises most frequently after or concomitantly with psoriasis, and since early diagnosis and treatment can prevent progression and disease-related disability, dermatologists need to be alert to its early changes and to be aware of its clinical and imaging characteristics.

Active PsA inflammation frequently involves the synovial membrane of finger and toe joints and/or tendons. US has recently been demonstrated to depict reliably the early changes and soft tissue abnormalities of inflammatory arthritis [25, 35–44]. Some authors have shown that it has acceptable specificity for overall joint pathology in affected and clinically unaffected hand and foot joints of patients with PsA, despite being less sensitive than magnetic resonance (MR); however, US is more widely available and economical [44]. 

In the majority of our patients with psoriasis and peripheral joint pain but no diagnosis of PsA, US showed findings consistent with synovitis and/or tenosynovitis in at least one finger and/or toe. X-ray evaluation disclosed structural damage in 11 patients who also had US abnormalities. While arthropathy was documented on standard radiographs in 11/36 patients, all the patients with US abnormalities, including those without pathological X-ray changes, had PsA confirmed by the rheumatologist. A larger number of soft tissue changes that were eventually diagnosed as PsA were found on US examination than on plain radiographs. These findings confirm previous reports of the ability of US to depict inflammatory and destructive changes in the fingers and toes of PsA patients [19, 44]. 

The majority of our patients who had abnormalities on US also had clear vascular spots in intra-articular and/or peritendinous spaces on PDS, reflecting an active inflammatory disease. In six Group A patients, the PDS signal was not judged to be related to PsA, given their normal morphological US and X-ray findings. Although a PDS signal at the level of the synovium is widely considered as a sign of hyperhemia, it is, in fact, also seen in some normal joints, especially when using highly sensitive devices. Terslev and coworkers found vascular spots in 11% of wrist and finger joints in 28 healthy subjects and concluded that PDS positivity per se should not be considered as a sign of inflammation. Since the synovial proliferation and/or effusion typical of chronic synovitis on gray-scale US may be undetectable in very early disease, a cut-off for vascular spots could be defined above which inflammation should be deemed to be present [45]. Such considerations may also explain the positive PDS findings seen in three Group B subjects who had increased vascularity on PDS. Thus, despite its high sensitivity, PDS lacks specificity in detecting diagnostic features of PsA and should be integrated with an accurate morphological US study, to demonstrate any inflammatory changes.

Finally, the psoriatic patients who were diagnosed with PsA and those in whom the disease was excluded had comparable demographic and clinical features (not shown), confirming that the cutaneous and the joint disease have independent courses. 

US proved valuable in detecting synovium abnormalities in the fingers and toes of patients with suspected PsA. The dermatologist, who usually manages psoriatic patients before the onset of the joint disease, should consider US to obtain an accurate assessment of suspicious findings, especially in presence of early changes. Although concomitant vascular evaluation by PDS did not seem to improve the ability of US to depict early PsA changes, the additional information on disease activity provided by PDS may be useful to assess response to therapy [31, 32]. 

Since US does not depict the specific changes of PsA, clinical and imaging findings suspicious for psoriatic joint disease should be assessed jointly by the dermatologist and the rheumatologist—whose cooperation is in our view always desirable—to classify psoriatic patients with joint abnormalities and to select the most appropriate treatment for both the skin and the joint disease. 

## Figures and Tables

**Table 1:** Clinical characteristics of the study population (mean ± SD).

	Group A (52 pts)	Group B (50 pts)
Age (years)	36.2 ± 9.6	32.1 ± 10.6
PASI	9.6 ± 11.5	7.9 ± 13.4
Mean duration of psoriasis (yrs)	10.4 ± 8.2	8.1 ± 7.5
Swollen joints (no.)	0.8 ± 2.1	0
Tender joints (no.)	2.3 ± 3.1	0

**Table 2:** Results of gray-scale US, PDS, X-rays, and rheumatological evaluation of 52 psoriatic patients with suspected psoriatic arthritis of fingers and/or toes.

	Imaging findings suspicious for PsA		
	US (tot 36)	PDS (tot 32)	X-rays (tot 11)
Patients diagnosed with PsA by the rheumatologist (tot 36)	36	29	11
Patients not diagnosed with PsA by the rheumatologist (tot 16)	0	6	0
